# Atherosclerosis and Bone Loss in Humans–Results From Deceased Donors and From Patients Submitted to Carotid Endarterectomy

**DOI:** 10.3389/fmed.2021.672496

**Published:** 2021-05-20

**Authors:** Diana Carmona-Fernandes, Sofia C. Barreira, Natacha Leonardo, Renata I. Casimiro, Alice M. Castro, Pedro Oliveira Santos, António N. Fernandes, Filipe Cortes-Figueiredo, Carolina A. Gonçalves, Rafael Cruz, Mariana L. Fernandes, Margarida Ivo, Luis M. Pedro, Helena Canhão, João Eurico Fonseca, Maria José Santos

**Affiliations:** ^1^Rheumatology Research Unit, Faculdade de Medicina, Instituto de Medicina Molecular João Lobo Antunes, Universidade de Lisboa, Centro Académico de Medicina de Lisboa, Lisboa, Portugal; ^2^Rheumatology Department, Centro Hospitalar Universitário Lisboa Norte, Hospital de Santa Maria, Lisboa, Portugal; ^3^Rheumatology Department, Hospital Garcia de Orta, Almada, Portugal; ^4^Instituto Português de Oncologia de Lisboa Francisco Gentil, Lisboa, Portugal; ^5^Pathology Department, Centro Hospitalar Universitário Lisboa Norte, Hospital de Santa Maria, Lisboa, Portugal; ^6^Faculdade de Medicina da Universidade de Lisboa, Centro Académico de Medicina de Lisboa, Instituto de Histologia e Biologia do Desenvolvimento, Lisboa, Portugal; ^7^Transplantation Department, Centro Hospitalar Universitário Lisboa Norte, Hospital de Santa Maria, Centro Académico de Medicina de Lisboa, Lisboa, Portugal; ^8^Vascular Surgery Department, Centro Hospitalar Universitário Lisboa Norte, Hospital de Santa Maria, Centro Académico de Medicina de Lisboa, Lisboa, Portugal; ^9^EpiDoC Unit-CEDOC, Comprehensive Health Research Center-CHRC, NOVA Medical School, Universidade NOVA de Lisboa, Lisboa, Portugal; ^10^Rheumatology Unit, Centro Hospitalar Universitario Lisboa Central, Lisboa, Portugal

**Keywords:** atherosclerosis, osteoporosis, pro inflammatory cytokines, bone remodeling biomarkers, tissue expression analysis

## Abstract

**Background and Aims:** Atherosclerosis and osteoporosis share common risk factors, as well as inflammatory mechanisms. Our aim was to understand how atherosclerotic lesions are related with disturbances in bone.

**Methods:** Gene expression of pro-inflammatory and bone metabolism related proteins (*IL-1*β*, IL-6, IL-17A, TNF, RANKL, OPG, COL1, CTSK, OCL, TRAP, CBFA1, DKK1, SOST, ADIPOQ*, and *ADIPOR1*) were analyzed in arteries and bones from 45 deceased donors and adipose tissue was used as control. Additionally, in 139 patients with advanced atherosclerosis submitted to carotid endarterectomy we compared calcium content (Alizarin red) and plaque inflammatory scores (CD3^+^, CD68^+^, and adiponectin) of patients with normal bone mineral density (BMD) with those with low BMD and explored the associations between gene expression in atherosclerotic plaques and BMD. Serum levels of pro-inflammatory and bone related proteins were measured both in donors and patients. Associations were investigated by the Pearson or Spearman correlation tests, and multivariate regression analyzes were performed when justified.

**Results:** Gene expression of bone remodeling and pro-inflammatory proteins correlated positively in bone and aorta, independently of age and sex of donors, but not in adipose tissue. The expression of bone formation genes was significantly higher in atheroma plaques from endarterectomized patients with normal vs. low BMD as well as inflammatory CD68^+^ scores, regardless of patients' age and sex, but not of body mass index. No relationship was observed between serum levels and gene expression levels of pro-inflammatory or bone remodeling proteins.

**Conclusions:** Our results suggest that the relationship between bones and vessels in the context of atherosclerotic disease and osteoporosis may rely on the intrinsic connection between the tissues involved, independently of disease stage. Serum measurements of pro-inflammatory and bone-remodeling proteins do not accurately translate tissue pathologic processes.

## Introduction

Atherosclerosis and osteoporosis are among the most prevalent diseases, frequently occurring in the same individual, and their prevalence increases with aging (1, 2).

Atherosclerosis is a chronic inflammatory process that evolves from fatty streaks to atheroma plaques and causes progressive stenosis of large and medium-sized arteries (3), as a consequence of accumulation of lipids, inflammatory cells, fibrous elements, cellular waste products and calcium (1).

Inflammation, as a key mechanism of atherosclerosis (2), affects its progression throughout all phases (4). Endothelial dysfunction and inflammatory lesions are mediated by several pro-inflammatory cytokines, present in atherosclerotic plaques produced by monocytes and macrophages (5). Infiltrates of CD68-positive macrophages and CD3- and CD8-positive T cells have been associated with plaque ruptures (6). Moreover, high serum levels of interleukin (IL)-1β, IL-6 and tumor necrosis factor (TNF) (3) are associated with an increase of cardiovascular (CV) risk, as demonstrated by epidemiological studies (7).

Osteoporosis (OP) is a skeletal bone disorder characterized by a decline in bone mineral density (BMD) and microarchitectural deterioration of bone tissue, which causes a reduction in bone strength and, consequently, leads to an increased risk of fracture (8–10). BMD can be determined by dual x-ray absorptiometry (DXA).

Bone is an active tissue, which is self-remodeled in a coupled action of bone-resorbing cells, osteoclasts, and bone forming cells, osteoblasts (11).

The Receptor Activator of NF-kB (RANK)/RANK Ligand (RANKL)/Osteoprotegerin (OPG) system, essential to the regulation of bone remodeling and to the physiopathology of OP (12), is closely related to inflammation. Not only inflammatory cells produce RANKL, but the interaction between RANK and RANKL leads to the release of pro-inflammatory cytokines, such as IL-1β, IL-6, and TNF, which increase bone resorption (13). Interestingly, the RANK/RANKL/OPG system and the Wnt pathway have also been implicated in the development of atherosclerosis and could be contributing pathways in the regulation of vascular calcification mechanisms (14).

These two diseases share common risk factors (1), as well as molecular and pathophysiological mechanisms (13), although conceivable common underlying mechanisms are not yet fully understood.

Our aim was to understand the relationship between atherosclerotic lesions and bone disturbances. Using samples from deceased donors, we aimed to analyze if a link between bone and vessel exists regarding gene expression patterns of pro-inflammatory cytokines and bone remodeling markers. Additionally, in a group of patients with advanced atherosclerosis submitted to carotid endarterectomy, we aimed to understand whether gene expression patterns of pro-inflammatory cytokines and bone remodeling markers in atherosclerotic plaques and plaque morphology are related to bone mineral density.

## Materials and Methods

### Patients

#### Deceased Donors' Samples

A sample of bone from the iliac crest and a section of the abdominal aorta were collected from 45 deceased donors at the time of organ collection for transplantation, immediately preserved at 4°C and processed on average in <24 h. A blood sample was also obtained. From a subgroup of seven patients (four men and three women), an additional sample of subcutaneous adipose tissue was collected. Due to confidentiality aspects, no clinical information beyond age and gender could be retrieved, but all of them had clearance to be organ donors, which means that they did not present major health issues at the time of death.

#### Endarterectomized Patients/Advanced Atherosclerosis Samples

Atherosclerotic plaques and fasting blood samples were collected from 139 patients submitted to carotid endarterectomy surgery. A structured protocol was applied to all patients for recording demographic data, CV risk factors, history of previous fractures, personal and family history of OP, other comorbidities, lifestyle, and past and current medication.

All patients performed a dual X-ray absorptiometry (DXA) and were classified with osteoporosis, osteopenia or normal BMD according to the WHO classification criteria (15).

This study was approved by the Ethics Committee of Centro Académico de Medicina de Lisboa and patients signed written informed consent prior to any protocol-specific procedure. All proceedings were conducted in accordance with the regulations governing biomedical investigation such as the Declaration of Helsinki, as amended in Fortaleza, Brazil (2013) (16).

### Biologic Samples Collection and Storage

Endarterectomy samples–the central and visually more developed plaque was sectioned crosswise over the longitudinal axis in two sections: one for RNA extraction that was fragmented in smaller pieces and immediately frozen in liquid nitrogen (snap-frozen) and stored at −80°C, and the other for histology and immunohistochemistry that was frozen in optimal cutting temperature compound (OCT) and stored at −80°C.

Deceased organ donor samples—Bone biopsies and aorta sections were processed the same way as the atheroma plaques.

Blood samples–were centrifuged upon arrival to the lab and the serum was collected and stored at −80°C for later analysis.

### RNA Isolation From Bones and Aortas

Samples were reduced to a fine powder with a pestle and mortar cold with liquid nitrogen and the RNA was extracted with TRIzol® (Invitrogen™), according to a modified version of Hughes et al. protocol (17). Briefly, the powder was placed in TRIzol® and homogenized, and chloroform was added to solubilize the lipids. A digestion with proteinase K was performed at 55°C, and it was subsequently treated with isopropyl alcohol to precipitate the RNA. The RNA pellet was cleaned with ethanol and then dissolved in RNase/DNase-free water.

The RNA quantification and quality were obtained by absorbance measured using the NanoDrop® ND- 1000 Spectrophotometer (NanoDrop Technologies, Inc., Wilmington, USA).

### RNA Expression–Quantitative RT-PCR

Total RNA was reverse-transcribed to cDNA according to the manufacturer's instructions (DyNAmo cDNA Synthesis Kit, Thermo Fisher Scientific Inc., Waltham, MA, USA).

The quantitative PCR was performed using DyNAmo Flash SYBR® Green qPCR Kit (Thermo Fisher Scientific Inc., Waltham, MA, USA) and the results measured with Rotor Gene 6000 (Qiagen, Germany) for deceased donors' samples and with 7500 Fast Real-Time PCR System (Applied Biosystems®, Foster City, CA, USA) for advanced atherosclerosis patients' samples. The sequences of the primers used (*IL-1*β*, IL-6, IL-17A, TNF, RANKL, OPG*, Collagen type I *(COL1)*, Cathepsin K *(CTSK)*, Osteocalcin (*OCL)*, Tartrate resistant acid phosphatase *(TRAP)*, Core-Binding Factor Alpha I *(CBFA1)*, Dickkopf-related protein 1 *(DKK1)*, Sclerostin *(SOST)*, Adiponectin (*ADIPOQ)*, Adiponectin receptor 1 (*ADIPOR1*) are listed in Supplementary Tables 1, 2. The efficiency of qPCR was analyzed using the standard curve method, as described previously (18). The values obtained were normalized with the housekeeping gene 18S rRNA.

### Histological Evaluation

Frozen plaques were sectioned crosswise over their longitudinal axis using a cryostat, and the major segment was used for histological analysis.

Alizarin Red S (Sigma, Missouri, USA) histological staining was used for calcium determination. The protocol was adapted (no de-paraffinization needed, slides were slowly immersed in distilled water) from IHC World website (19).

Immunohistochemical staining of the plaques to identify a proinflammatory/unstable profile was also performed with CD3 (eBioscience, San Diego, USA), CD68 (eBioscience, San Diego, USA) and Adiponectin (Boster Biological Technology, Pleasanton, USA) antibodies. Tissue sections were incubated with the primary antibody and with EnVision+ (Dako, Glostrup, Denmark). Color was developed in solution containing diaminobenzidine-tetrahydrochloride (Sigma, Missouri, USA), 0.5% H_2_O_2_ in phosphate-buffered saline buffer (pH 7.6). Slides were counterstained with hematoxylin and mounted (20).

Histological and immunohistochemical evaluations were performed using a semi-quantitative score of 0 to 3 (0–0 to 10% staining; 1–10 to 50% staining; 2–50 to 75% staining; 3–more than 75% staining). Slides were observed in a ZEISS Primo Star (ZEISS, Oberkochen, Germany) microscope.

### Serum Cytokine and Bone Markers Quantification

RANKL and OPG serum levels were determined using Biomedica ELISA (Enzyme-Linked Immunosorbent Assay) (Cat. No. BI-20462 and BI-20403, respectively), and C-terminal telopeptide of type 1 collagen (CTX) and procollagen type 1 N propeptide (P1NP) with SunRed Biological Technology (Cat. No. 201-12-1350 and 201-12-2130, respectively). Data were acquired in the microplate reader Infinite® M200 (Tecan).

Cytometric Bead Array (CBA) determination was performed for pro-inflammatory cytokines, IL-1β, IL-6, IL-17A, and TNF [BD™ CBA Enhanced Sensitivity Flex Set; Cat. No. 561509 (for IL-1β), Cat. No. 561512 (for IL-6), Cat. No. 562143 (for IL-17A), and Cat. No. 561516 (for TNF)] and data were collected in the Accuri™ C6 flow cytometer from BD™ Biosciences.

### Statistical Analysis

Statistical analysis was performed using IBM SPSS version 20. Quantitative variables are described as means and standard deviation and qualitative variables as percentages and absolute frequencies.

Statistical significance was considered for a two-tailed *p* < 0.05 and the confidence interval for all statistical analyses was 95%. The normality of the distribution for continuous variables was evaluated using Kolmogorov–Smirnov test. Comparisons between groups and correlations were performed using parametric and non-parametric tests, as appropriate. When justified, multivariable linear regression analyses with backward selection of covariates was performed.

## Results

### Deceased Donors

#### Population Description

A total of 45 donors were included in this study with ages ranging between 15 and 80 years old: 23 men with 49.6 ± 17.8 years old and 22 women with 61.1 ± 12.9 years old. The aortas were evaluated macroscopically and in five of them (11.1%) calcifications were visible. These patients corresponded to a 78-year-old man and four women with a mean age of 66.5 ± 9.9 years old.

#### RNA Expression–Quantitative RT-PCR

**Figure 1:**
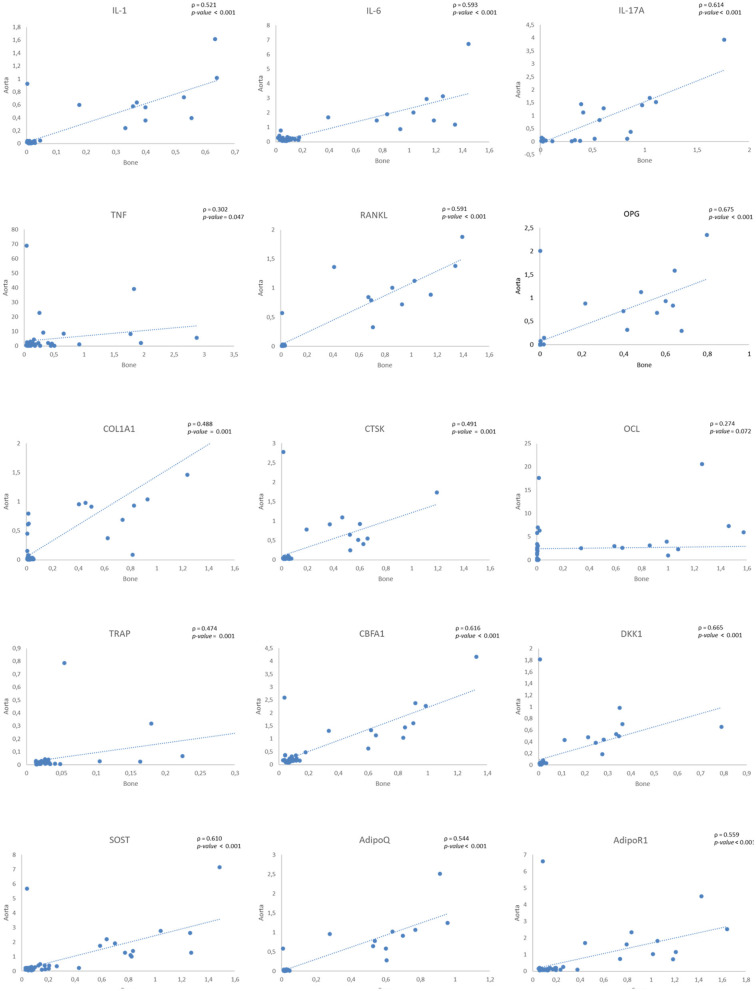
Correlations of gene expression levels between bone and aorta samples. IL, Interleukin; TNF, Tumor necrosis factor; RANKL, Receptor Activator of NF-kB Ligand; OPG, Osteoprotegerin; COL1A1, Collagen type I; CTSK, Cathepsin K; OCL, Osteocalcin; TRAP, Tartrate resistant acid phosphatase; CBFA1, Core-Binding Factor Alpha I; DKK1, Dickkopf-related protein 1; SOST, Sclerostin; AdipoQ, Adiponectin; AdipoR1, Adiponectin receptor 1.

A positive correlation between gene expression levels in bone and aorta samples was observed for all studied genes, except *OCL* gene (see Figure 1). The correlations found were classified, as described by Evans (21), as weak for the *TNF* gene; moderate for the *IL-1*β*, IL-6, RANKL, COL1A1, CTSK, TRAP, ADIPOQ*, and *ADIPOR1* genes; and strong for the IL*-17A, OPG, CBFA1, DKK1*, and *SOST* genes.

No association was found between gene expression levels of bone and aorta samples individually with adipose tissues samples.

Donors with macroscopic aortic calcifications had higher expression of *RANKL* and *TRAP* in the aorta and of *IL-17A* and *SOST* in bone—Supplementary Table 3.

No significant differences in the gene expression of inflammatory and bone remodeling markers in aorta, bone or adipose tissue samples were found between men and women—Supplementary Table 4 neither in association with age.

#### Serum Cytokines and Bone Markers Analysis

We found that IL-1β levels were significantly higher in men than in women [442.7 fg/mL (range 179.3–4569) vs. 89.4 fg/mL (range 38.9–504.9); *p* = 0.011]. Age did not influence serum cytokine or bone markers levels.

No significant correlations were found between serum levels and gene expression levels, either from bone, aorta or adipose tissue samples, for any of the proteins studied.

### Atherosclerosis Patients

#### Population Description

A total of 139 patients with advanced atherosclerosis were included in the study, where 95 (68.3%) were men, 70.3 ± 8.7 years old, and 44 (31.7%) were women, 71.5 ± 9.6 years old.

We have further compared the clinical characteristics, co-morbidities and therapies between patients with normal BMD (t-score >−1) and patients with low BMD (t-score ≤ −1). Details are listed in Table 1. Patients with low BMD were older (*p* = 0.001) and had lower BMI levels (*p* < 0.001). They did not differ significantly in any of the other variables evaluated.

**Table 1:** Clinical characteristics, co-morbidities and therapies of atherosclerosis patients with normal and low BMD.

**Characteristic**	**Normal BMD (*N* = 69)**	**Low BMD (*N* = 64)**	***p*-value**
Age (years)	67.9 ± 8.6	73.0 ± 8.6	0.001
Gender	49 M/20 F	41 M/23 F	0.392
Alcohol (above 3U/day)	19 (27.5%)	15 (23.4%)	0.554
Calcium intake (mg/day)	848.6 ± 498.27	896.8 ± 468.9	0.372
Current/Previous smokers	9 (13.0%)/28 (40.6%)	8 (12.5%)/29 (45.3%)	0.850
Active lifestyle	7 (10.1%)	5 (7.8%)	0.724
BMI (Kg/m^2^)	28.5 ± 4.4	25.5 ± 4.0	<0.001
Hypertension	59 (85.5%)	49 (76.6%)	0.187
Previous CV event	44 (63.8%)	37 (57.8%)	0.482
Dyslipidemia	59 (85.5%)	53 (82.8%)	0.833
Type 2 Diabetes	20 (28.9%)	17 (26.6%)	0.716
Glucocorticoids (> 3 months)	4 (5.8%)	8 (12.5%)	0.167
Statins therapy	52 (75.4%)	46 (71.9%)	0.651

*Quantitative variables are presented as means ± SD and qualitative variables as absolute values and proportions of total (%). BMD, bone mineral density; BMI, body mass index; CV, cardiovascular*.

#### RNA Expression–Quantitative RT-PCR

Regarding bone remodeling markers in the atheroma plaques, we found that genes associated with bone formation were expressed at higher levels in patients with normal BMD than in patients with low BMD (Table 2). The difference was statistically significant for, specifically, *CBFA1* (0.98 ± 0.08 vs. 0.71 ± 0.06, *p* = 0.009) and *OCL* (1.27 ± 0.12 vs. 0.83 ± 0.08, *p* = 0.003). The association between BMD and *CBFA1* and *OCL* expression remained significant in a linear regression model adjusted for gender, age, and BMI.

**Table 2:** Gene expression levels in the atheroma plaques of atherosclerosis patients with normal and low BMD.

**Gene**	**Normal BMD (*N* = 69)**	**Low BMD (*N* = 64)**	***p*-value**
IL-1β	1.86 ± 1.57	1.59 ± 1.18	0.617
IL-6	0.65 ± 0.35	0.60 ± 0.31	0.527
IL-17A	1.62 ± 1.56	1.93 ± 1.95	0.360
TNF	137.2 ± 172.2	112.2 ± 175.6	0.262
RANKL	1.46 ± 0.95	1.23 ± 0.91	0.137
OPG	2.66 ± 1.99	2.29 ± 1.82	0.288
RANKL/OPG	0.46 ± 0.26	0.44 ± 0.28	0.767
COL1A1	0.89 ± 0.54	0.72 ± 0.48	0.065
CTSK	0.81 ± 0.49	0.77 ± 0.50	0.477
OCL	1.27 ± 0.84	0.84 ± 0.54	0.009
TRAP	6.93 ± 10.51	5.69 ± 7.54	0.442
CBFA1	0.98 ± 0.63	0.71 ± 0.47	0.015
DKK1	3.63 ± 2.67	3.04 ± 2.66	0.116
SOST	2.89 ± 1.89	2.89 ± 2.17	0.647
AdipoQ	34.4 ± 47.1	27.2 ± 35.6	0.671
AdipoR1	1.91 ± 1.16	1.62 ± 1.01	0.223

*Variables are presented as means ± SD. BMD, bone mineral density; IL, Interleukin; TNF, Tumor Necrosis Factor; RANKL, Receptor Activator of NF-kB Ligand; OPG, Osteoprotegerin; COL1A1, Collagen type I; CTSK, Cathepsin K; OCL, Osteocalcin; TRAP, Acid phosphatase tartrate resistant; CBFA1, Core-Binding Factor Alpha I; DKK1, Dickkopf-related protein 1; SOST, Sclerostin; AdipoQ, Adiponectin; AdipoR1, Adiponectin receptor 1*.

Additionally, we found that *OCL* gene expression level in the plaques was higher in patients with dyslipidemia (1.12 ± 0.73 vs. 0.74 ± 0.69, *p* = 0.007). However, in multivariate analysis *OCL* gene expression levels remained significantly associated only with BMD.

The gene expression of inflammatory markers (*IL-1*β*, IL-6, IL-17A*, and *TNF*) in the plaques was similar in patients with normal and low BMD and was not related to other patient's characteristics (demographic, BMI, lifestyle habits, co-morbidities or medication).

#### Histological Evaluation

Plaque CD3 and CD68 immunohistochemistry scores were higher in patients with normal BMD than in patients with low BMD (Table 3). Additionally, CD3 (0.82 ± 0.97 vs. 0.34 ± 0.53, *p* = 0.006) and CD68 (0.89 ± 0.89 vs. 0.45 ± 0.63, *p* = 0.005) immunohistochemistry scores were higher in male patients. In the independent analysis, CD3 (ρ = −0.221, *p* = 0.009) and CD68 (ρ = −0.181, *p* = 0.033) immunohistochemistry scores were inversely related to patients age.

**Table 3:** Histological scores of atherosclerosis patients with normal and low BMD.

**Molecule**	**Normal BMD (*N* = 69)**	**Low BMD (*N* = 64)**	***p*-value**
Alizarin Red S	1.94 ± 0.95	1.92 ± 1.04	0.991
CD3^+^	0.78 ± 0.91	0.47 ± 0.76	0.020
CD68^+^	0.91 ± 0.84	0.55 ± 0.79	0.004
Adiponectin	1.16 ± 0.90	1.13 ± 0.90	0.780

*Variables are presented as means ± SD. BMD, bone mineral density; CD, Cluster of differentiation*.

In a linear regression model adjusted for sex and age, CD68 scores were significantly associated with BMD (β = −0.203, *p* = 0.016). In a model with further adjustment to BMI, only gender and BMI maintained a significant association with CD68 scores.

No significant correlations were found between any of the histological studies performed (alizarin red S, CD3, CD68, or adiponectin) and the results of the ELISAs or gene expression quantifications.

#### Serum Cytokines and Bone Markers Analysis

Regarding serum levels analyzed, we did not find differences between patients with normal or low BMD (Table 4).

**Table 4:** Serum levels of atherosclerosis patients with normal and low BMD.

**Protein**	**Normal BMD (*N* = 69)**	**Low BMD (*N* = 64)**	***p*-value**
IL-1β (fg/mL)	14.8 ± 49.1	12.9 ± 35.8	0.780
IL-6 (fg/mL)	2139.5 ± 1730.7	1982.3 ± 1953.7	0.235
IL-17A (fg/mL)	17.9 ± 50.2	15.3 ± 30.8	0.380
TNF (fg/mL)	11.8 ± 47.7	8.02 ± 37.2	0.352
RANKL (pmol/L)	0.022 ± 0.019	0.019 ± 0.015	0.389
OPG (pmol/L)	8.11 ± 5.45	7.97 ± 5.44	0.885
RANKL/OPG	0.0033 ± 0.0040	0.0034 ± 0.0040	0.990
CTX (ng/mL)	30.8 ± 6.9	30.8 ± 8.3	0.865
P1NP (ng/mL)	130.5 ± 75.2	135.6 ± 75.4	0.678
CTX/P1NP	0.21 ± 0.09	0.23 ± 0.08	0.288
Adiponectin (ng/mL)	114909.4 ± 93591.1	130745.4 ± 99158.3	0.403

*Variables are presented as means ± SD. BMD, bone mineral density; IL, Interleukin; TNF, Tumor Necrosis Factor; RANKL, Receptor Activator of NF-kB Ligand; OPG, Osteoprotegerin; CTX, C-terminal telopeptide of type 1 collagen; P1NP, procollagen type 1 N propeptide*.

No significant associations were found between gene expression in the plaque and serum levels of bone remodeling markers or inflammatory markers.

## Discussion

With our work we aimed to understand how atherosclerotic lesions are related with disturbances in bone and specifically if there is a role for inflammation in this relationship.

Regarding several genes related to inflammation and bone remodeling we found that, at the gene expression level, there was a positive correlation between bones and vessels, specifically with the aorta, suggesting a link between these two systems. In addition, the observed correlation did not extend to adipose tissue, which supports that this is not a widespread finding.

No differences on the gene expression pattern with age or sex were found in deceased donors' samples, suggesting that the relationships described above do not vary significantly throughout life, neither between women and men, pointing that, in pathologic processes, bone and vessel disturbances are possibly linked due to an intrinsic connection between the tissues involved. To the best of our knowledge there are no previous reports of any relation between the expression of these genes, either in the vessels or in bones, with age or gender.

We could not find an association between gene expression and the circulating levels of the same proteins. As reviewed by Vogel et al. (22) 60% of the variation in protein concentration that cannot be explained by measuring mRNAs alone is at least partially due to translation and protein degradation. Other works have also observed the lack of association between OPG, PTH, and RANKL gene expression in bone and serum levels (23, 24).

In the second part of our work we have used atherosclerotic plaques from patients with advanced atherosclerosis that were submitted to endarterectomy surgery and where bone mineral density was evaluated by DXA. We found that more than 45% of the enrolled patients had low BMD, which is in accordance with the age range of this population and with previous studies where bones and vessels were both evaluated by imaging methods (25, 26).

We found that the expression of bone formation genes (*CBFA1* and *OCL*) on atherosclerotic plaques was lower in those patients who have decreased BMD. Yet, our results do not suggest lower risk of plaque calcification, as we have not found any differences in the calcium content between patients with normal or low BMD.

The presence of these proteins in plaques is in accordance with previous studies showing that calcified plaques composition share some features of bone structure (27), but these results seem contradictory to other studies (28–30) that reported an association between low BMD and vascular calcification. These differences might be related to different methodologies used to quantify vascular calcification (microscopic vs. macroscopic) and also to different stages of plaque development, as calcification can begin at any point of plaque formation and progression (27).

When analyzing the results of immunohistochemical staining performed on the atherosclerotic plaques, our results support that atheroma plaques from men have higher levels of inflammation, as previously proposed (31, 32). BMI also influences plaques inflammatory score, highlighting the link between obesity and atherosclerosis progression (33). Accordingly, in deceased donors, serum levels of pro-inflammatory cytokine IL-1β were also higher in men.

Patients with low BMD had plaques with lower inflammatory profile. Low BMD has been associated with echogenic (more calcified) plaques (34, 35) and calcification and inflammation are thought to be active at different stages of disease progression (36), with inflammation at early phases and calcification predominantly later (32, 37), which might explain our findings.

Despite the limitations related to the cross-sectional design, correlative nature of the work with lack of functional data, and the lack of clinical information of deceased donors, our study has some strengths. Samples from deceased donors allowed us to directly evaluate gene expression on bone and aortas from the same subjects and in a wide range of ages, going behind indirect diagnostic methods used *in vivo*. Regarding atherosclerosis patients, we did not have access to bone tissue of these patients to perform the study directly on the affected tissue. However, contrary to most studies, we had access to carotid atheroma plaques, and not only to ultrasound evaluation of atherosclerosis, and all patients had BMD evaluated by DXA.

## Conclusions

We have described a positive correlation between bone and aorta concerning the expression of inflammation and bone remodeling genes, independent of age and sex. Bone-remodeling genes are not only expressed on atherosclerotic plaques, but also on vessel walls, regardless of the presence of plaques.

Additionally, the atheroma plaques of patients with low BMD present lower levels of bone formation markers (*CBFA1* and *OCL*) and a lower score CD68 immunostaining than those with normal BMD.

Our results suggest that the relationship between the changes observed in vessels and bones in the context of atherosclerotic disease and OP may rely on the intrinsic connection between the tissues involved, independently of the disease stage.

## Data Availability Statement

The original contributions presented in the study are publicly available upon request. This data can be found here: https://zenodo.org/record/1403777#.YD0JGi0qK9Y.

## Ethics Statement

The studies involving human participants were reviewed and approved by Ethics Committee of Centro Académico de Medicina de Lisboa. The patients/participants provided their written informed consent to participate in this study.

## Author Contributions

DC-F, MS, JF, and HC contributed to conception and design of the study. SB, AC, PS, AF, FC-F, CG, RCr, MF, MI, and LP collected clinical data and biologic samples. NL, RCa, and DC-F performed laboratory work. DC-F organized the database and performed the statistical analysis. DC-F and SB wrote the manuscript. All authors contributed to manuscript revision, read, and approved the submitted version.

## Conflict of Interest

The authors declare that the research was conducted in the absence of any commercial or financial relationships that could be construed as a potential conflict of interest.
