# Supplementary material for: Atherosclerosis and Bone Loss in Humans–Results From Deceased Donors and From Patients Submitted to Carotid Endarterectomy
Source: Front Med (Lausanne). 2021 May 20;8:672496. doi: 10.3389/fmed.2021.672496 (PMC8172790; doi:10.3389/fmed.2021.672496)
Supplement: Supplementary file 1 [file Data_Sheet_1.PDF]

S1 Table. Housekeeping and target genes primer sequences used for deceased donors' samples

Gene	Forward primer sequence	Reverse primer sequence
18S rRNA	GGAGTATGGTTGCAAAGCTGA	ATCTGTCAATCCTGTCCGTGT
IL-1β	TACCTGTCCTGCGTGTTGAA	TCTTTGGGTAATTTTTGGGATCT
IL-6	GATGAGTACAAAAGTCCTGATCCA	CTGCAGCCACTGGTTCTGT
IL-17A	TTCCCCCGGACTGTGATGGTCA	CAGGGTCCTCATTGCGGTGGAGA
TNF	CAGCCTCTTCTCCTTCCTGAT	GCCAGAGGGCTGATTAGAGA
RANKL	AGAGAAAGCGATGGTGGATG	TATGGGAACCAGATGGGATG
OPG	CGCTCGTGTTTCTGGACAT	GTAGTGGTCAGGGCAAGGG
COL1A1	ACGAAGACATCCCACCAATC	AGATCACGTCATCGCACAAC
CTSK	GCCAGACAACAGATTTCCATC	CAGAGCAAAGCTCACCACAG
OCL	CCAGGCAGGTGCGAAG	TCAGCCAACTCGTCACAGTC
TRAP	CGGCCACGATCACAATCT	GCTTTGAGGGGTCCATGA
CBFA1	CGGAATGCCTCTGCTGTTA	TCTGTCTGTGCCTTCTGGGT
DKK1	CAGGCGTGCAAATCTGTCT	AATGATTTTGATCAGAAGACACACATA
SOST	AGACCAAAGACGTGTCCGAG	GGGATGCAGAGGAAGTC
AdipoQ	GGTGAGAAGGGTGAGAAAGA	TTTCACCGATGTCTCCCTTAG
AdipoR1	TTGTGTACAAGGTCTGGGAGG	GATGCTCTTGAAGCAAGCCC

IL – Interleukin; TNF – Tumor necrosis factor; RANKL - Receptor Activator of NF-kB Ligand; OPG – Osteoprotegerin; COL1A1 – Collagen type I; CTSK – Cathepsin K; OCL – Osteocalcin; TRAP – Tartrate resistant acid phosphatase; CBFA1 - Core-Binding Factor Alpha I; DKK1 - Dickkopf-related protein 1; SOST - Sclerostin; AdipoQ - Adiponectin; AdipoR1 - Adiponectin receptor 1.
