# Supplementary material for: Atherosclerosis and Bone Loss in Humans–Results From Deceased Donors and From Patients Submitted to Carotid Endarterectomy
Source: Front Med (Lausanne). 2021 May 20;8:672496. doi: 10.3389/fmed.2021.672496 (PMC8172790; doi:10.3389/fmed.2021.672496)
Supplement: Supplementary file 3 [file Data_Sheet_3.PDF]

S3 Table. Gene expression levels in aorta, bone and adipose tissues in donors with and without macroscopic aortic calcifications

Gene	Donors with macroscopic aortic calcifications (n=5)			Donors without macroscopic aortic calcifications (n=40)			p-value		
	Aorta	Bone	Adipose tissue	Aorta	Bone	Adipose tissue	Aorta	Bone	Adipose tissue
**IL-1ß**	0.40±0.34	0.22+.23	0.50±0.06	0.16±0.34	0.10±0.19	0.40±0.14	0.151	0.178	0.262
**IL-6**	1.42±1.18	0.54±0.52	1.37±0.82	0.57±1.18	0.26±0.41	1.06±0.19	0.133	0.169	0.588
**IL-17A**	0.94±0.80	**0.59±0.43**	1.09±0.71	0.29±0.73	**0.20±0.38**	0.89±0.17	0.072	**0.039**	0.672
**TNF**	3.62±3.09	0.32±0.23	2.31±1.98	4.71±12.7	0.36±0.63	1.06±1.02	0.851	0.908	0.319
**RANKL**	**0.71±0.64**	0.46±0.47	0.86±0.37	**0.20±0.43**	0.19±0.39	0.73±0.26	**0.023**	0.152	0.607
**OPG**	0.59±0.54	0.26±0.27	0.65±0.19	0.23±0.55	0.11±0.23	0.55±0.17	0.177	0.165	0.348
**COL1A1**	0.70±0.65	0.53±0.54	0.79±0.23	0.38±0.94	0.23±0.61	0.94±0.30	0.465	0.303	0.517
**CTSK**	0.55±0.44	0.25±0.24	0.54±0.15	0.24±0.54	0.14±0.26	0.44±0.06	0.22	0.382	0.291
**OCL**	2.88±2.65	1.99±2.91	2.78±1.67	6.57±25.44	0.18±0.41	1.94±0.61	0.749	0.236	0.389
**TRAP**	**0.33±0.33**	0.10±0.19	0.29±0.25	**0.08±0.22**	0.06±0.12	0.22±0.09	**0.031**	0.475	0.746
**CBFA1**	1.13±0.89	0.46±0.40	0.98±0.30	0.53±0.84	0.21±0.32	0.65±0.09	0.139	0.113	0.081
**DKK1**	0.32±0.29	0.16±0.15	0.28±0.04	0.17±0.34	0.07±0.16	0.27±0.05	0.339	0.258	0.734
**SOST**	1.27±1.11	**0.63±0.32**	1.59±0.83	0.76±1.45	**0.26±0.39**	1.14±0.44	0.453	**0.046**	0.396
**AdipoQ**	0.59±0.51	0.33±0.33	0.52±0.16	0.22±0.48	0.15±0.27	0.46±0.09	0.115	0.167	0.538
**AdipoR1**	1.24±1.02	0.52±0.41	1.44±0.50	0.57±1.28	0.31±0.42	1.15±0.22	0.272	0.283	0.348

IL – Interleukin; TNF – Tumor necrosis factor; RANKL - Receptor Activator of NF-kB Ligand; OPG – Osteoprotegerin; COL1A1 – Collagen type I; CTSK – Cathepsin K; OCL – Osteocalcin; TRAP – Tartrate resistant acid phosphatase; CBFA1 - Core-Binding Factor Alpha I; DKK1 - Dickkopf-related protein 1; SOST - Sclerostin; AdipoQ - Adiponectin; AdipoR1 - Adiponectin receptor 1.
