# Supplementary material for: Atherosclerosis and Bone Loss in Humans–Results From Deceased Donors and From Patients Submitted to Carotid Endarterectomy
Source: Front Med (Lausanne). 2021 May 20;8:672496. doi: 10.3389/fmed.2021.672496 (PMC8172790; doi:10.3389/fmed.2021.672496)
Supplement: Supplementary file 4 [file Data_Sheet_4.PDF]

S4 Table. Gene expression levels in aorta, bone and adipose tissues in donors, according to gender

Gene	Males (n=23)			Females (n=22)			p-value		
	Aorta	Bone	Adipose tissue	Aorta	Bone	Adipose tissue	Aorta	Bone	Adipose tissue
**IL-1ß**	0.26±0.43	0.13±0.21	0.42±0.14	0.12±0.22	0.09±0.17	0.48±0.10	0.206	0.504	0.576
**IL-6**	0.84±1.51	0.33±0.45	1.03±0.23	0.47±0.72	0.25±0.41	1.41±0.78	0.305	0.514	0.497
**IL-17A**	0.48±0.93	0.26±0.44	0.82±0.19	0.26±0.54	0.22±0.36	1.19±0.65	0.356	0.621	0.323
**TNF**	6.03±14.89	0.25±0.39	1.42±0.99	3.15±8.26	0.46±0.75	1.84±2.28	0.431	0.272	0.750
**RANKL**	0.31±0.53	0.26±0.45	0.75±0.26	0.21±0.42	0.18±0.36	0.83±0.38	0.495	0.519	0.738
**OPG**	0.38±0.70	0.15±0.26	0.55±0.07	0.16±0.33	0.09±0.21	0.66±0.19	0.181	0.443	0.313
**COL1A1**	0.36±0.62	0.19±0.31	0.96±0.24	0.47±1.15	0.33±0.81	0.76±0.21	0.676	0.446	0.364
**CTSK**	0.38±0.68	0.19±0.31	0.44±0.07	0.17±0.26	0.11±0.19	0.54±0.14	0.179	0.292	0.243
**OCL**	10.11±33.38	0.21±0.41	2.10±0.48	2.03±2.27	0.56±1.54	2.57±1.84	0.260	0.307	0.701
**TRAP**	0.14±0.29	0.08±0.15	0.30±0.14	0.08±0.20	0.05±0.06	0.13±0.03	0.407	0.257	0.195
**CBFA1**	0.72±1.03	0.27±0.37	0.72±0.12	0.46±0.61	0.20±0.29	0.84±0.41	0.318	0.505	0.681
**DKK1**	0.23±0.43	0.10±0.19	0.27±0.04	0.13±0.21	0.07±0.12	0.28±0.05	0.313	0.450	0.891
**SOST**	1.08±1.85	0.31±0.43	1.19±0.45	0.55±0.70	0.29±0.37	1.53±0.87	0.213	0.870	0.526
**AdipoQ**	0.32±0.59	0.21±0.32	0.52±0.11	0.20±0.36	0.12±0.23	0.44±0.14	0.450	0.339	0.456
**AdipoR1**	0.91±1.64	0.39±0.47	1.15±0.23	0.37±0.60	0.28±0.35	1.44±0.50	0.154	0.325	0.337

IL – Interleukin; TNF – Tumor necrosis factor; RANKL - Receptor Activator of NF-kB Ligand; OPG – Osteoprotegerin; COL1A1 – Collagen type I; CTSK – Cathepsin K; OCL – Osteocalcin; TRAP – Tartrate resistant acid phosphatase; CBFA1 - Core-Binding Factor Alpha I; DKK1 - Dickkopf-related protein 1; SOST - Sclerostin; AdipoQ - Adiponectin; AdipoR1 - Adiponectin receptor 1.
